# Supplementary material for: Dietary salt with nitric oxide deficiency induces nocturnal polyuria in mice via hyperactivation of intrarenal angiotensin II-SPAK-NCC pathway
Source: Commun Biol. 2022 Feb 28;5:175. doi: 10.1038/s42003-022-03104-6 (PMC8885931; doi:10.1038/s42003-022-03104-6)

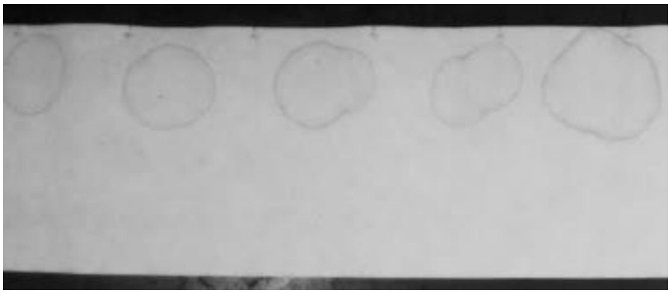

Supplementary Figure 1. **Urine spots in aVSOP method.** A laminated filter paper pre-treated to turn the edge of urine stains deep purple when exposed to urine, was used in the aVSOP method.

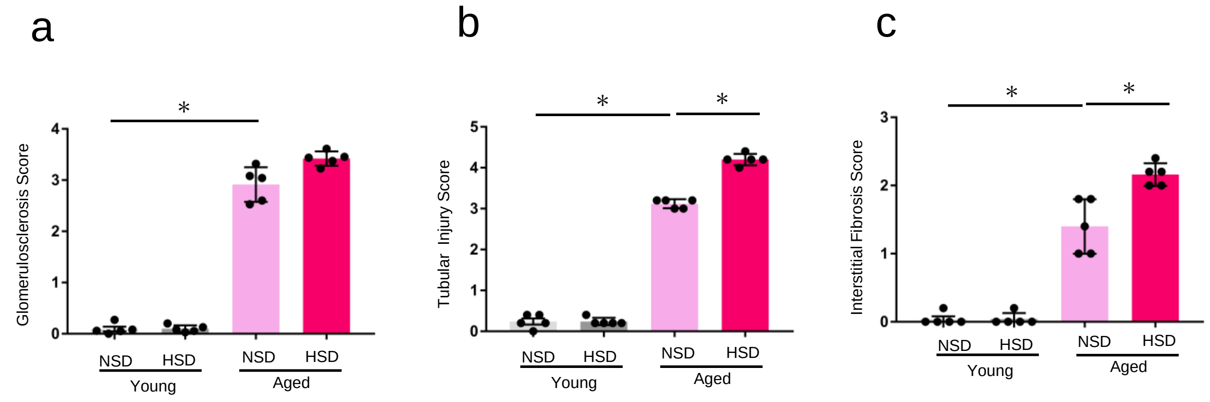

Supplementary Figure 2. Histological quantification of the effect of salt loading on the kidney in a young mice and aged mice. a. Glomerulosclerosis score, **b.** Tubular injury score, **c.** Interstitial fibrosis score Data are expressed as the mean ± SEM, * $P<0.05$ (n = 5 mice per group). Statistical analysis was performed using a two-tailed Student's *t*-test.

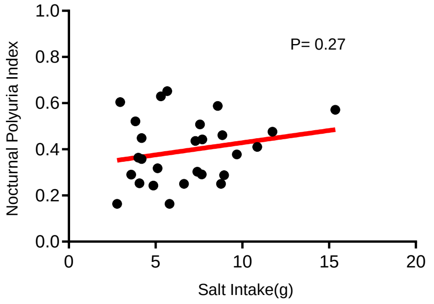

Supplementary Figure 3. Correlation between dietary salt intake and nocturnal polyuria index in all subjects (n=27).

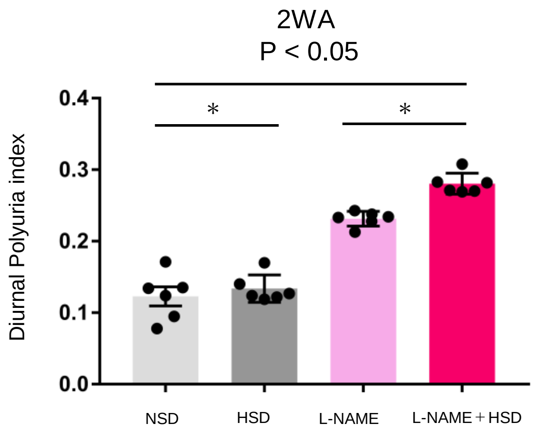

Supplementary Figure 4. DPi in each group. Data are expressed as the mean ± SEM, * P<0.05 (NSD, HSD, L-NAME, L-NAME+HSD: n=6). Statistical analysis was performed using the two-way ANOVA or the Tukey-Kramer method.

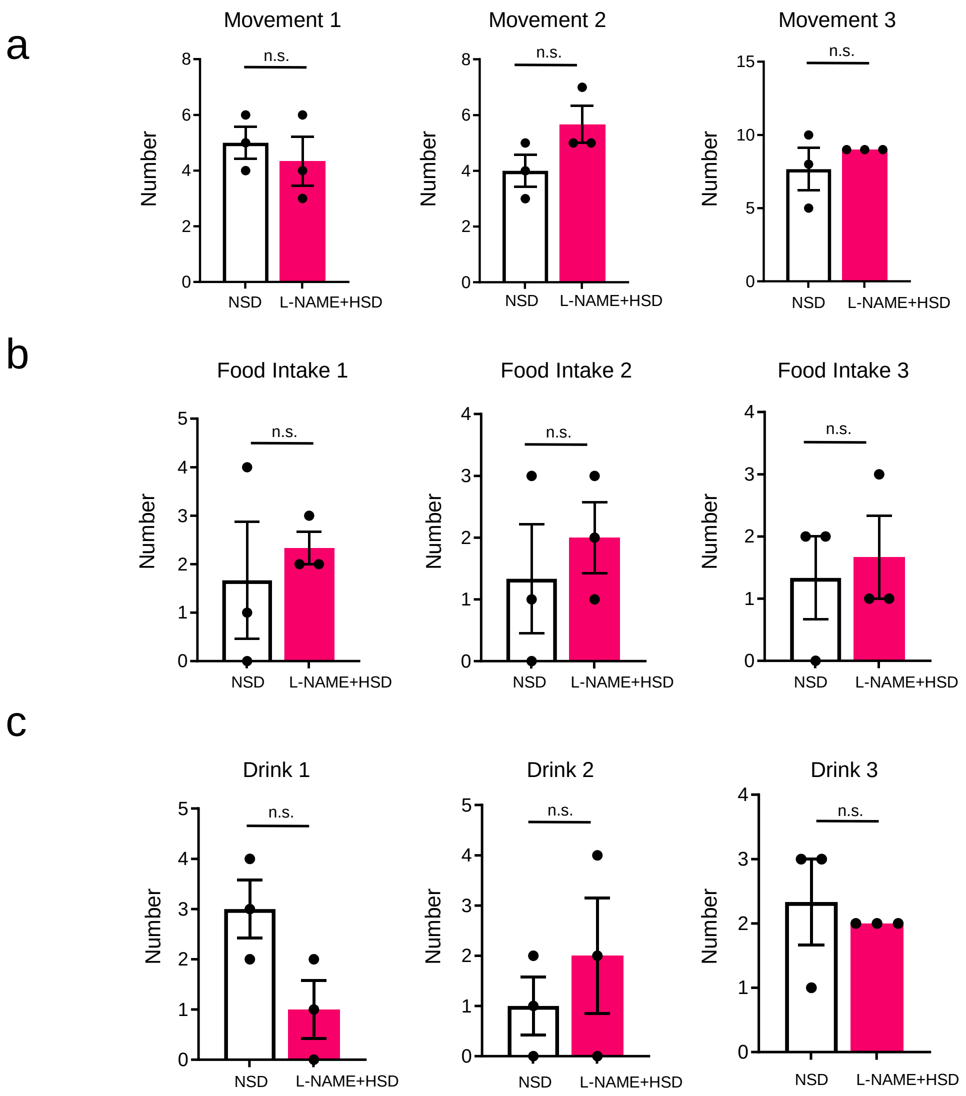

Supplementary Figure 5. Salt loading and L-NAME administration do not alter behavioral patterns. **a.** Number of movements; **b.** Number of food intakes; **c.** Number of drinks. The inactive period was divided into three equal parts (1:ZT0-ZT4, 2:ZT4-ZT8, 3:ZT8-ZT12), and each behavioral pattern was assessed in 1-h videos for the NSD and L-NAME+HSD groups. Data are expressed as the mean ± SEM, * $P<0.05$ ($n = 3$ mice per group). Statistical analysis was performed using a two-tailed Student's *t*-test.

	All patients (n=27)	Low NO (n=13)	High NO (n=14)
Age, yr	61 (42-79)	57 (44-74)	65 (42-79)
Sex (M/F), no.	10/17	7/6	3/11
Nocturnal Polyuria Index	0.39 (0.16-0.65)	0.40 (0.24-0.59)	0.39 (0.16-0.65)
serum Cr, mg/dL	0.71 (0.47-1.20)	0.77 (0.53-1.20)	0.65 (0.47-1.01)
eGFR, ml/min/1.73m^2	75.4 (48.6-98.8)	73.5 (48.6-94.5)	77.1 (56.5-98.58)
NOx, μmol/Cr mg	0.29 (0.02-0.58)	0.17 (0.02-0.25)	0.39 (0.26-0.58)
Urinary salt excretion, g	6.8 (2.8-15.4)	8.2 (4.0-15.4)	5.5 (2.8-8.8)

Supplementary Table 1. Characteristics of 27 patients. Data are expressed as median (range), or number.

	active period	inactive period	daily
NaV (mEq)			
NSD	0.11	0.01	0.12
HSD	0.19	0.02	0.21
L-NAME	0.10	0.02	0.12
L-NAME+HSD	0.16	0.06	0.22
UV (μL)			
NSD	1945	275	2220
HSD	2194	336	2530
L-NAME	1451	439	1890
L-NAME+HSD	1668	655	2323
[Na] (mEq/L)			
NSD	57	36	54
HSD	87	60	83
L-NAME	69	46	64
L-NAME+HSD	96	92	95

Supplementary Table 2. Sodium excretion, urine volume, and estimated sodium concentration during the active and inactive periods and 24 h. Na excretion was calculated using the metabolic cage, and urine volume was calculated via the aVSOP method, as it is more reliable than the metabolic cage in measuring minute urine volumes. The estimated Na concentrations were calculated from these results.

	active period	inactive period	daily
NaV (mEq)			
NP model	0.16	0.06	0.22
NP model+HCTZ	0.24	0.04	0.28
UV (μL)			
NP model	1668	655	2323
NP model+HCTZ	2338	552	2890
[Na] (mEq/L)			
NP model	96	92	95
NP model+HCTZ	103	72	97

Supplementary Table 3. The effect of an NCC inhibitor (HCTZ) on sodium excretion, urine volume, and estimated sodium concentration during active and inactive periods and 24 h. NP: nocturnal polyuria

Supplementary Figure 6: Unedited blot images

Active period

pNCC

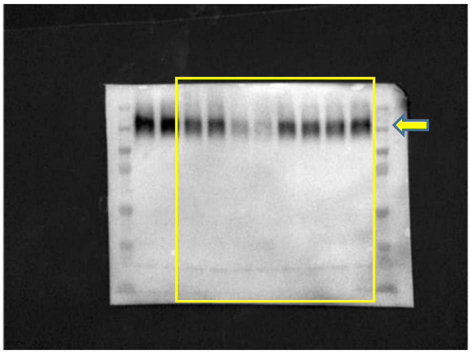

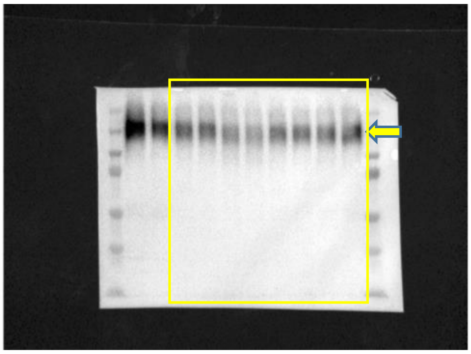

NCC

αENaC

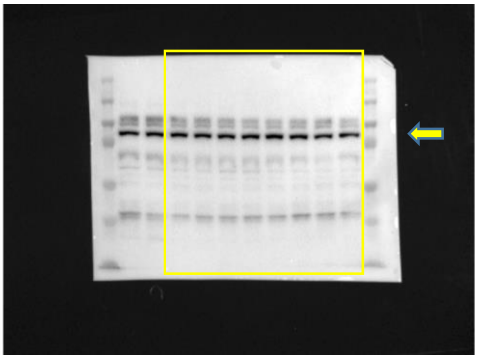

ACTN

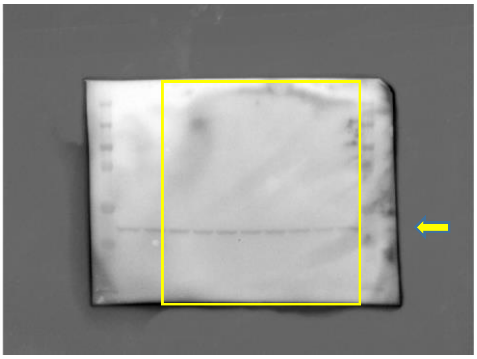

unedited gel

Active period

pNCC

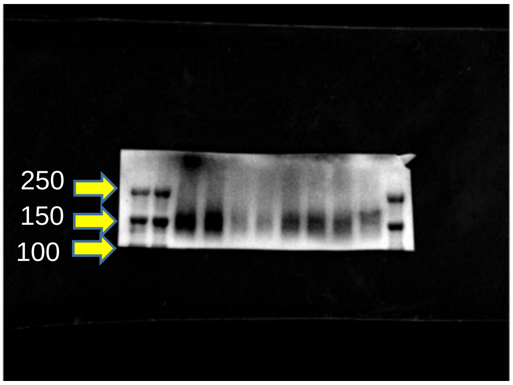

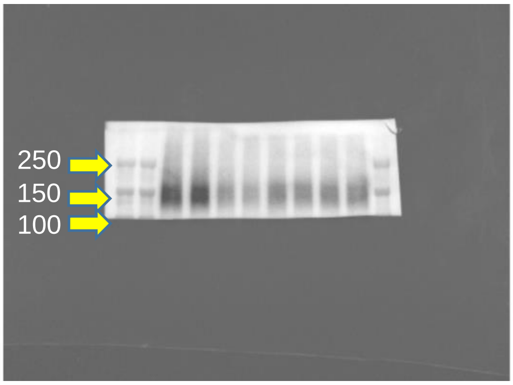

NCC

αENaC

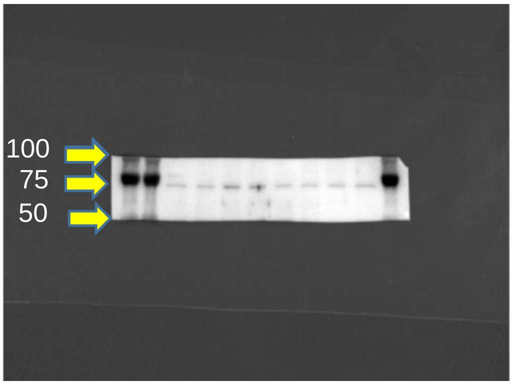

ACTN

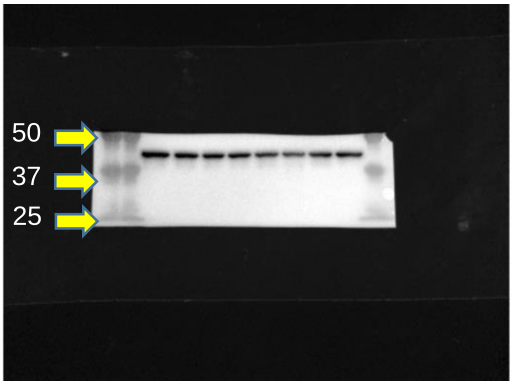

unedited gel

Active period

βENaC

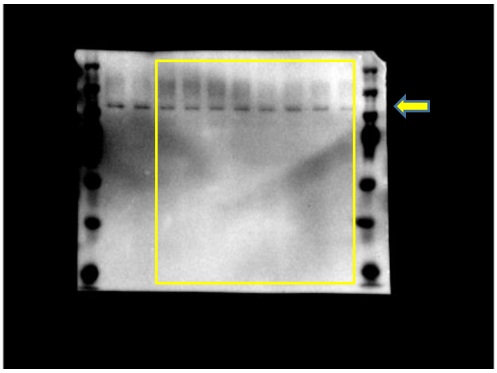

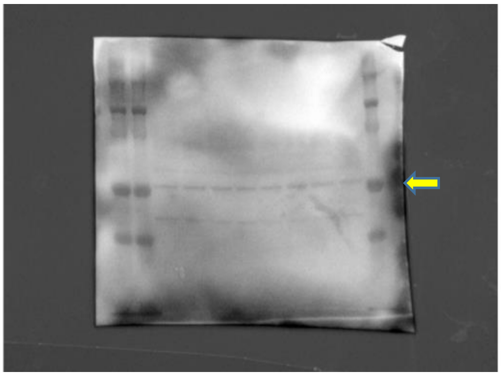

γENaC

ACTN

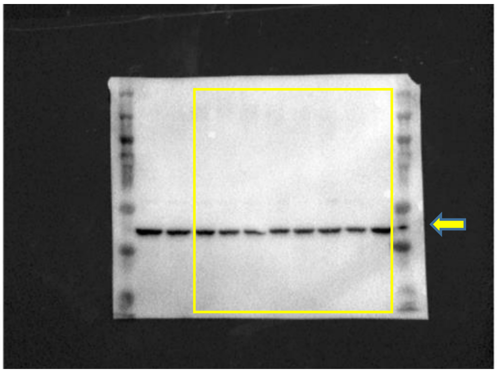

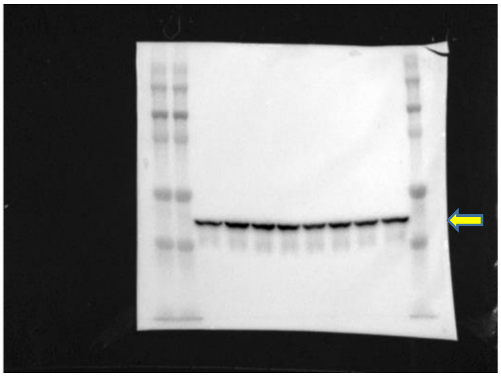

ACTN

unedited gel

Active period

αENaC

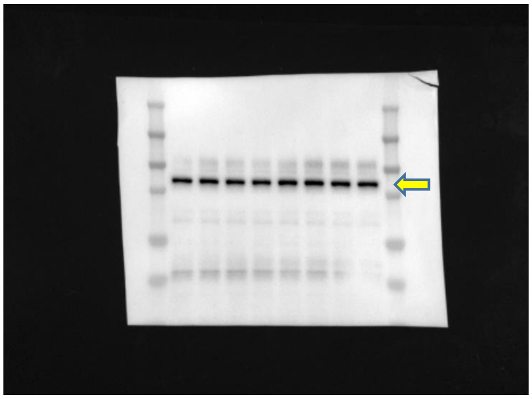

βENaC

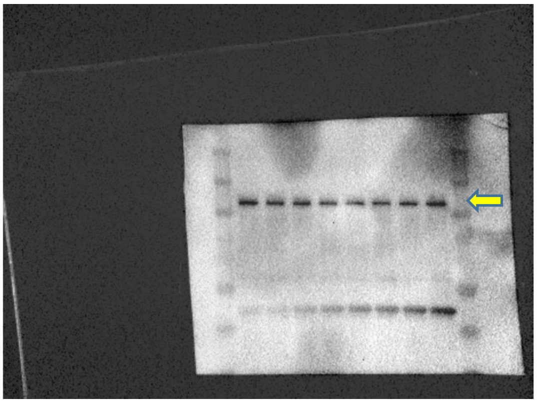

γENaC

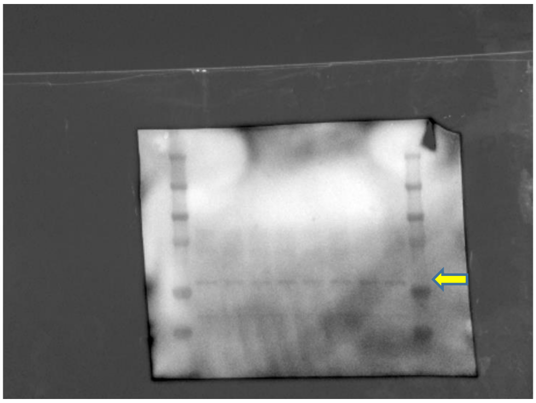

ACTN

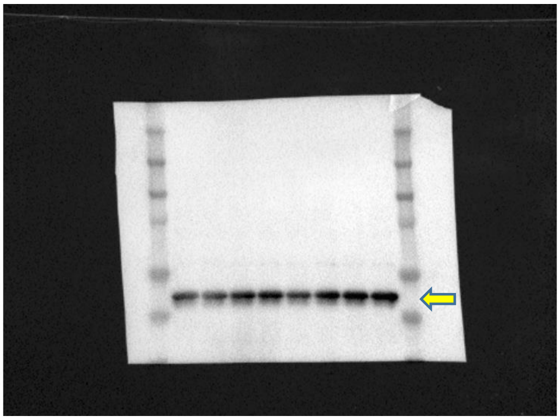

unedited gel

Active period

pSPAK

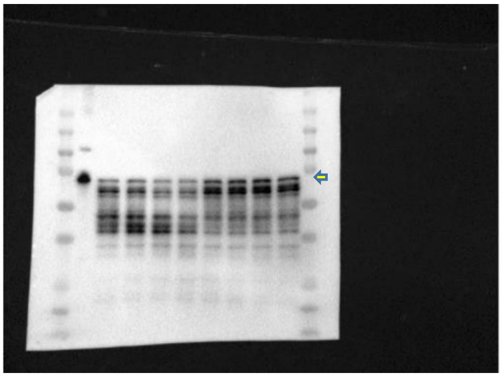

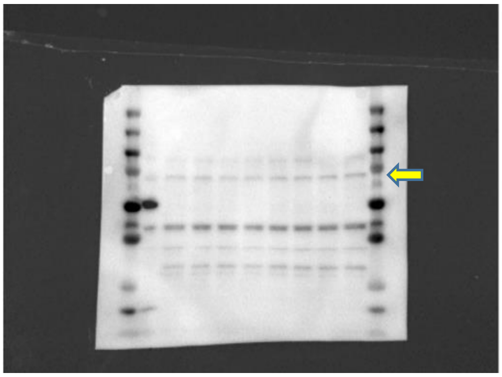

SPAK

ACTN

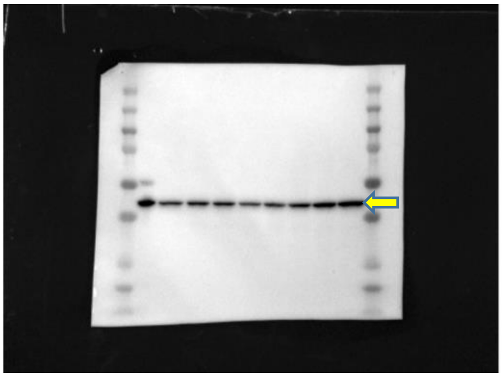

unedited gel

Active period

AGT

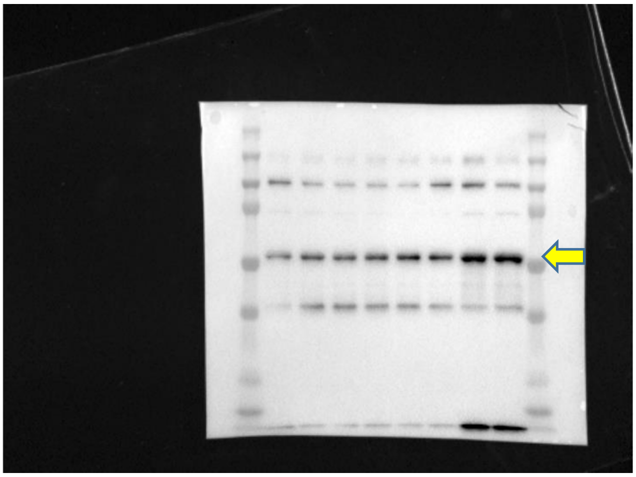

ACTN

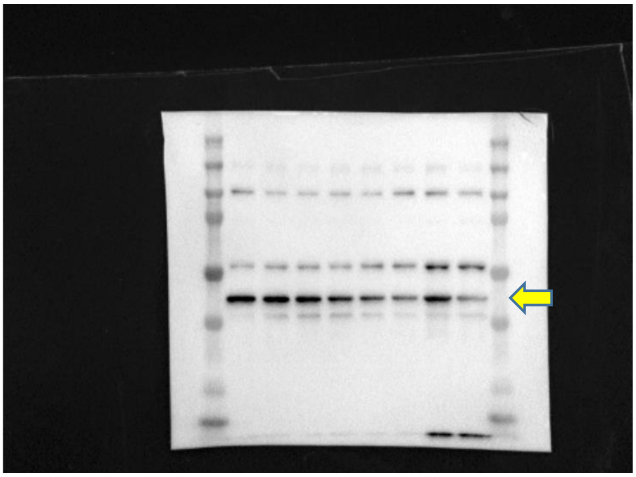

Supplement: Supplementary file 2 — Supplementary Information [file 42003_2022_3104_MOESM2_ESM.pdf]
