# Supplementary material for: Dietary salt with nitric oxide deficiency induces nocturnal polyuria in mice via hyperactivation of intrarenal angiotensin II-SPAK-NCC pathway
Source: Commun Biol. 2022 Feb 28;5:175. doi: 10.1038/s42003-022-03104-6 (PMC8885931; doi:10.1038/s42003-022-03104-6)
Supplement: Supplementary file 3 — Description of Additional Supplementary Files [file 42003_2022_3104_MOESM3_ESM.pdf]

Description of Additional Supplementary Files

File name: Supplementary Data 1
Description: Source data for the graphs in the main figures.
