# Supplementary material for: Dietary salt with nitric oxide deficiency induces nocturnal polyuria in mice via hyperactivation of intrarenal angiotensin II-SPAK-NCC pathway
Source: Commun Biol. 2022 Feb 28;5:175. doi: 10.1038/s42003-022-03104-6 (PMC8885931; doi:10.1038/s42003-022-03104-6)
Supplement: Supplementary file 5 — Reporting Summary [file 42003_2022_3104_MOESM5_ESM.pdf]

Corresponding author(s): Hiroshi Kiuchi
Last updated by author(s): Jan 7, 2022

# Reporting Summary

Nature Portfolio wishes to improve the reproducibility of the work that we publish. This form provides structure for consistency and transparency in reporting. For further information on Nature Portfolio policies, see our Editorial Policies and the Editorial Policy Checklist.

## Statistics

For all statistical analyses, confirm that the following items are present in the figure legend, table legend, main text, or Methods section.

| n/a | Confirmed | |
|---|---|---|
| ☐ | ☒ | The exact sample size (*n*) for each experimental group/condition, given as a discrete number and unit of measurement |
| ☐ | ☒ | A statement on whether measurements were taken from distinct samples or whether the same sample was measured repeatedly |
| ☐ | ☒ | The statistical test(s) used AND whether they are one- or two-sided<br>*Only common tests should be described solely by name; describe more complex techniques in the Methods section.* |
| ☒ | ☐ | A description of all covariates tested |
| ☒ | ☐ | A description of any assumptions or corrections, such as tests of normality and adjustment for multiple comparisons |
| ☐ | ☒ | A full description of the statistical parameters including central tendency (e.g. means) or other basic estimates (e.g. regression coefficient) AND variation (e.g. standard deviation) or associated estimates of uncertainty (e.g. confidence intervals) |
| ☐ | ☒ | For null hypothesis testing, the test statistic (e.g. *F*, *t*, *r*) with confidence intervals, effect sizes, degrees of freedom and *P* value noted<br>*Give P values as exact values whenever suitable.* |
| ☒ | ☐ | For Bayesian analysis, information on the choice of priors and Markov chain Monte Carlo settings |
| ☒ | ☐ | For hierarchical and complex designs, identification of the appropriate level for tests and full reporting of outcomes |
| ☒ | ☐ | Estimates of effect sizes (e.g. Cohen's *d*, Pearson's *r*), indicating how they were calculated |

*Our web collection on statistics for biologists contains articles on many of the points above.*

## Software and code

Policy information about availability of computer code

| | |
|---|---|
| Data collection | n/a |
| Data analysis | JMP (SAS Institute, Cary, NC, USA) or GraphPad Prism 8.0 (GraphPad Software, SD, CA, USA). |

For manuscripts utilizing custom algorithms or software that are central to the research but not yet described in published literature, software must be made available to editors and reviewers. We strongly encourage code deposition in a community repository (e.g. GitHub). See the Nature Portfolio guidelines for submitting code & software for further information.

## Data

Policy information about availability of data

All manuscripts must include a data availability statement. This statement should provide the following information, where applicable:
- Accession codes, unique identifiers, or web links for publicly available datasets
- A description of any restrictions on data availability
- For clinical datasets or third party data, please ensure that the statement adheres to our policy

All relevant data are available from

# Field-specific reporting

Please select the one below that is the best fit for your research. If you are not sure, read the appropriate sections before making your selection.

☒ Life sciences ☐ Behavioural & social sciences ☐ Ecological, evolutionary & environmental sciences

For a reference copy of the document with all sections, see nature.com/documents/nr-reporting-summary-flat.pdf

# Life sciences study design

All studies must disclose on these points even when the disclosure is negative.

| | |
|---|---|
| Sample size | n/a |
| Data exclusions | No data was exculed |
| Replication | Representative experiments have been repeated at least two times. |
| Randomization | n/a |
| Blinding | Urine volume measurements(aVSOP methods) were performed by two or three blind investigator. |

# Reporting for specific materials, systems and methods

We require information from authors about some types of materials, experimental systems and methods used in many studies. Here, indicate whether each material, system or method listed is relevant to your study. If you are not sure if a list item applies to your research, read the appropriate section before selecting a response.

## Materials & experimental systems

| n/a | Involved in the study | |
|---|---|---|
| ☐ | ☒ | Antibodies |
| ☒ | ☐ | Eukaryotic cell lines |
| ☒ | ☐ | Palaeontology and archaeology |
| ☐ | ☒ | Animals and other organisms |
| ☐ | ☒ | Human research participants |
| ☒ | ☐ | Clinical data |
| ☒ | ☐ | Dual use research of concern |

## Methods

| n/a | Involved in the study | |
|---|---|---|
| ☒ | ☐ | ChIP-seq |
| ☒ | ☐ | Flow cytometry |
| ☒ | ☐ | MRI-based neuroimaging |

## Antibodies

**Antibodies used**

Fluorescent immunostaining
rabbit anti-NCC (1:100, #AB3553, Millipore)
rabbit anti-phosphorylated NCC (threonine 53, 1:1000, #p1311-53, Phospho Solution).
Anti-rabbit antibody with Alexa Fluor 568 (1:300, #A11011, Thermo Fisher Scientific)
anti-rabbit antibody with Alexa Fluor 488 (1:300, #A11034, Thermo Fisher Scientific) was used as the secondary antibody.

Immunoblotting
rabbit anti-SPAK (1:500, #AB79045, Abcam)
rabbit anti-phosphorylated SPAK (1:1000, #07-2273, Millipore),
rabbit anti-NCC (1:1000, #AB3553, Millipore)
rabbit anti-phosphorylated NCC (threonine 53, 1:1000, #p1311-53, Phospho Solution)
rabbit anti-ENaCα (1:1000, #SPC-403, Stresmark Biosciences, Inc.)
rabbit anti-angiotensinogen antibody (1:1000, #AB213705, Abcam)
Anti-rabbit IgG HRP antibody (1:5000, #7074, Cell Signaling Technology) was used as the secondary antibody.

**Validation**

rabbit anti-SPAK (1:500, #AB79045, Abcam)was validated by mice brain by ourselves and by Abcam.
rabbit anti-phosphorylated SPAK (1:1000, #07-2273, Millipore)is validated for use in WB for the detection by Millipore.
rabbit anti-NCC (1:1000, #AB3553, Millipore)was validated by rat kidney as an positive control by ourselves and Abcam.
abbit anti-phosphorylated NCC (threonine 53, 1:1000, #p1311-53, Phospho Solution)was validated by rat kidney and DOCA hypertension mice kidney by ourselves.
rabbit anti-ENaCα (1:1000, #SPC-403, Stresmark Biosciences, Inc.)was validated by rat kidney by ourselves and Stressmark Biosciences.
rabbit anti-angiotensinogen antibody (1:1000, #AB213705, Abcam)was validated by mice heart by ourselves and Abcam.

## Animals and other organisms

Policy information about studies involving animals; ARRIVE guidelines recommended for reporting animal research

| | |
|---|---|
| Laboratory animals | 19week or 80week -old C57BL6/J male |
| Wild animals | No wild animals were used in this study. |
| Field-collected samples | No field-collected samples were used in this study. |
| Ethics oversight | The mouse work was performed under the study protocol (No. J006580-013) as approved by the Institutional Animal Care and Use Committee. |

Note that full information on the approval of the study protocol must also be provided in the manuscript.

## Human research participants

Policy information about studies involving human research participants

| | |
|---|---|
| Population characteristics | Healthy kidney transplant donors (n=27) were included in the study.<br>male 10 female 17 ,age42-79(median61) |
| Recruitment | Every Healthy kidney transplant donors who consented to our study was recruited. |
| Ethics oversight | the Institutional Review Board No. 18418 |

Note that full information on the approval of the study protocol must also be provided in the manuscript.
